# Effects of different ascorbic acid doses on the mortality of critically ill patients: a meta-analysis

**DOI:** 10.1186/s13613-019-0532-9

**Published:** 2019-05-20

**Authors:** Ying Wang, Huan Lin, Bing-wen Lin, Jian-dong Lin

**Affiliations:** 1 Clinical School, First Affiliated Hospital of Fujian Medical University, Fuzhou, China; 2 Shool of Mathematics and Statistics, University of New South Wales, Sydney, Australia; 3 Department of Intensive Care Unit, First Affiliated Hospital of Fujian Medical University, Chazhong Road, Fuzhou, Fujian Province China

**Keywords:** Ascorbic acid, Critical illness, Sepsis, Septic shock, Burn

## Abstract

**Background:**

Low levels of ascorbic acid (AA) have been detected in critically ill patients in which AA supplementation leads to promising outcomes. However, the ability of AA to reduce mortality in critically ill patients remains controversial. In this study, we have performed a meta-analysis to evaluate the effects of AA dose on the mortality of critically ill adults.

**Methods:**

Electronic databases were searched for trials in which AA had been intravenously administered to critically ill patients regardless of the dose or the co-administration of antioxidant agents. The predefined primary outcome included all-cause mortality at final follow-up.

**Results:**

The included trials enrolled a total of 1210 patients. Intravenous (IV) AA doses of 3–10 g/d reduced the mortality of critically ill patients (OR 0.25; 95% CI (0.14–0.46); *p* < 0.001; *I*^2^ = 0.0%), while low (< 3 g/d) and high AA doses (≥ 10 g/d) had no effect (OR 1.44; 95% CI (0.79–2.61); *p* = 0.234; *I*^2^ = 0.0% versus OR 1.12; 95% CI (0.62–2.03); *p* = 0.700; *I*^2^ = 0.0%). AA was associated with a decreased duration of vasopressor support and mechanical ventilation, but did not influence fluid requirement or urine output during the first 24 h of admission. The number of patients suffering from acute kidney injury and the length of intensive care unit or hospital stays were also unaffected by the AA.

**Conclusion:**

Intravenous AA reduces the duration of vasopressor support and mechanical ventilation; 3–10 g AA results in lower overall mortality rates. Given the limitations of the primary literature, further studies are required to fully clarify the effectiveness of AA during the management of critically ill patients.

**Electronic supplementary material:**

The online version of this article (10.1186/s13613-019-0532-9) contains supplementary material, which is available to authorized users.

## Background

Ascorbic acid (AA) is a water-soluble vitamin and an essential endogenous trace element that scavenges reactive oxygen species (ROS) [1, 2] and reduces immunosuppression [3]. Previous studies have shown that patients with critical illness, particularly sepsis, have low levels of AA in the plasma [4–7] which holds prognostic value due to its inverse correlation with multiple organ failure [7]. Given the low levels of AA in critically ill patients, supplemental AA has been administered to animal models of sepsis [8–13] and intensive care unit (ICU) patients [4, 14–24, 30–48]. Results from these studies suggest that AA improves the condition of critically ill patients. Its beneficial effects include the attenuation lipid peroxidation, reduced vascular permeability, low levels of microvascular dysfunction, the preservation of endothelial function and microcirculatory flow, improved endogenous vasopressor synthesis, increased vasopressor sensitivity, and hemodynamic stability. This ultimately leads to reduced organ injury and dysfunction in critically ill patients.

Despite promising preliminary results, the benefits of AA remain controversial. Marik and coworkers [20] stated that AA as part of a “cocktail” therapy can reduce mortality in critically ill patients (8.5% vs. 40.4%, *p* < 0.01), while Lin et al. [19] reported no significant effects of AA infusion (26% vs. 23%, *p* = 0.8). Notably, the dose of AA varied between these studies, which may account for the discrepancies. Furthermore, the small sample size and single-center nature of the studies questions their reproducibility.

In this review, we provide a comprehensive meta-analysis (MA) of all studies in which AA has been intravenously administered to critically ill patients. We aimed to identify whether the dose of AA impacts mortality and other clinical parameters in this setting, including resuscitation fluid requirement, urine output, acute kidney injury (AKI), vasopressor requirement, the duration of mechanical ventilation, and the length of ICU and/or hospital stay.

## Materials and methods

This study was performed and prepared according to the guidelines proposed by Cochrane Collaboration in the Cochrane Handbook for Systematic Reviews of Interventions (http://www.cochrane handbook.org) and Preferred Reporting Items for Systematic Reviews and Meta-Analyses (PROSMA) statement [25, 26].

### Search strategy

We searched articles of all languages published from inception to November 2018 in PubMed, Embase, Ovid and the Cochrane Central Register of Controlled Trials using the following keywords along with MeSH terms: “ascorbic acid” and “sepsis” or “critical illness” or “Intensive Care Unit” or “burn”. We collected all studies in which AA was intravenously administered to adult patients with critical illness.

### Study selection criteria

The following trails were included

1. Performed on adults with critical illness.
2. Intravenous AA regardless of the dose vs. placebo or no-intervention.
3. Primary outcome was mortality at the final follow-up.

The following trails were excluded

1. Performed on children.
2. AA administrated orally or enterally.
3. Lack of mortality data.

### Data extraction

Data were independently extracted by the first and third authors. Extracted data consisted of the first author name, year of publication, type of the study, study population, number of patients, AA dose, antioxidant agent, treatment initiation, treatment duration, mortality at follow-up, and other clinical parameters. We resolved disagreements through discussions until a consensus was reached.

### Outcome measurements and definitions

The primary outcome was all-cause mortality at final follow-up. Secondary outcomes included resuscitation fluid requirement, urine output, patients suffering from AKI, vasopressor requirement, duration of mechanical ventilation, and length of ICU and/or hospital stay.

### Assessment of risk of bias

We used the Cochrane Collaboration tool to assess the risk of bias in randomized controlled trials (RCTs) [26, 27]. Domains containing random sequence generation (selection bias), allocation concealment (selection bias), blinding of participants and personnel (performance bias), blinding of the outcome assessment (detection bias), incomplete outcome data (attrition bias), selective reporting (reporting bias), and other bias were assessed. The remaining observational trials were assessed using the ROBINS-I tool [28]. Domains include bias due to confounding, bias in selection of participants into the study, bias in classification of interventions, bias due to deviation from intend intervention, bias due to missing data, bias in measurement of outcomes, and bias in selection of the reported results. We rated each domain of the trials as low risk, unclear, or high risk. Trials were considered low risk when each independent domain was rated as low risk. Any domain rated as unclear or high risk increased the overall risk score.

### Statistical analysis

Data were analyzed using Statistics/Data Analysis 15.1. The results of dichotomous data were presented as forest plots through the odds ratios (ORs) with 95% confidence intervals (CIs). Forest plots using SMD with 95% CI were performed for the assessment of continuous data. We quantified heterogeneity via the *I*^2^ statistic. Data were pooled through random (M-H heterogeneity) models if the value of *I*^2^ was greater than 50%, regarded as heterogeneity [29]. A *p* value ≤ 0.05 was considered statistically significant, except when otherwise specified.

## Results

### Literature search

**Fig. 1:**
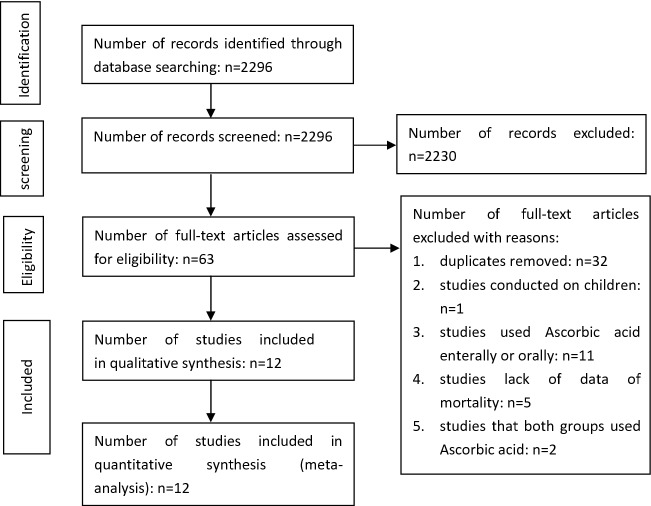
Study flow diagram chart

Through database searches (Fig. 1), 2296 records were identified. We screened both titles and abstracts according to the inclusion and exclusion criteria, leaving 63 studies that were deemed suitable for inclusion. After reviewing the full texts, 12 studies [14–24, 36] were finally included (Table 1). Studies were excluded for the following reasons: duplication (*n* = 32), performed on children (*n* = 1) [30], enteral administration of AA (*n* = 11) [31–35, 37–42], lack of mortality data (*n* = 5) [4, 43–46], both groups administrated AA (*n* = 2) [47, 48].

**Table 1:** The main characteristics of the studies included

Author	Year	Design	Patients(n intervention/n control)	Ascorbic acid dose	Other antioxidant	Time of treatment initiation	Duration of treatment	Mortality at final follow-up (*N* intervention/*N* control)	Other clinical parameters
Ferrón-Celma	2009	RCT	Undergoing abdominal surgery and postoperative mortality risk > 30% (10/10)	450 mg/d	None	12 h post-surgery	From 12 h post-surgery to d6 post-surgery	6 days: 6/4	Caspase-3↓, PARP proteins↓ and Bcl-2 levels ↑in treatment group
Fowler III	2014	RCT	Severe sepsis (16: high dose 8 and low dose 8)/8	50 or 200 mg/kg/d	None	2–4 h following randomization	96 h	28 days: 7(low dose: 3, high dose 4)/5	SOFA scores↓(high dose was the most remarkable); CRP ↓in treatment group; PCT↓ only in the high-dose group; The TM↑ in control group, while treatment group remained the same.
Zabet	2016	RCT	Post-operation patients with septic shock and need a vasopressor (14/14)	25 mg/kg q6 h	None	Unavailable	72 h	28 days: 2/9	Less vasopressor
Kahn	2011	Observational	Burn covering greater than 20% of TBSA (17/16)	66 mg/kg/hr	None	At a mean time of 52 ± 26 min after admission	Unavailable	Hospital mortality: 4/3	Mean PaO2/FiO2 during the study period: ascorbic acid group: 201.32 ± 32.22; control group: 221.23 ± 13.87
Tanaka	2000	Quasi-RCT	Burn covering greater than 30% of TBSA (19/18)	66 mg/kg/h	None	After admission	The initial 24-h	Hospital mortality: 9/7	Edema ↓ body weight gain ↓ in treatment group
Lin	2018	Observational	During burn shock resuscitation (38/42)	66 mg/kg/h	None	Mean time: 4.01 ± 15 h	Unavailable	Hospital mortality: 10/10	Acute renal failure↑ in high-dose group
Marik	2017	Observational	Severe sepsis or septic shock and a PCT level ≥ 2 ng/ml (47/47)	1.5 g q6 h	None	Unavailable	4 days	Hospital mortality: 4/19	The median 72-h PCT ↓,the 72-h ⊿SOFA score ↓in treatment group
Razmkon	2011	RCT	GCS ≤ 8 with diffuse axonal injury(49/27)	500 mg/d or 10 g/d	None	Within 24 h	7 days	Hospital mortality: 14 (low dose: 7, high dose: 7)/8	No promise for short- and long-term neurological outcome in treatment group
Sandesc	2017	Observational	Injury severity score > 16(35/32)	3000 mg/d	*N*-Acetylcysteine 1200 mg/d	Unavailable	Unavailable	Hospital mortality: 5/11	Possibility to develop into Sepsis or MODS↓;at discharge/until death the APACHE II score ↓
Palli	2017	RCT	Needs for contrast-enhanced CT(60/64)	2 g/d	*N*-Acetylcysteine 1200 mg/d	2 h before and at 10 and 18 h after contrast agent	18 h	Hospital mortality: 15/11	Failed to reduce the incidence of CIN;
Galley	1997	Quasi-RCT	Septic shock and need a vasopressor(16/14)	1 g/d	*N*-Acetylcysteine and vitamin E	Unavailable	Unavailable	Hospital mortality: 11/8	Beneficial hemodynamic changes.
Nathens	2002	RCT	Undergoing general surgery/trauma (301/294)	1000 mg q8 h	α-Tocopherol 1000 IU q8 h	At a mean time of 11.3 ± 6 h	28 days	28 days: 4/7	Multiple organ failure↓, concentrations of TNF-α, IL-1β, IL-6↓, ICU and hospital stay ↓,ventilator-free days ↑ in treatment group.

A quasi-RCT uses quasi-random method of allocating participants to different interventions, such as allocation by date of birth, day of the week, medical record number, month of the year, or the order in which participants are included in the study

### Study characteristics

Of the 12 included trials, eight were RCTs and four were retrospective studies. All studies were published from 1997 to 2018. The characteristics of each trial are shown in Table 1. The studies recruited patients with severe sepsis or septic shock [15, 16, 20, 24], burn shock [17–19], critical injury [21, 22], post-operation [14, 16, 36], trauma [36] and those in need of contrast-enhanced CT in the ICU [23]. The sample sizes ranged from 20 to 595. The included studies enrolled a total of 1210 patients of which 624 were administrated IV AA, and 586 were control subjects. The dose of AA ranged from 450 mg/d to 66 mg/kg/h.

### Risk of bias and quality of evidence

**Fig. 2:**
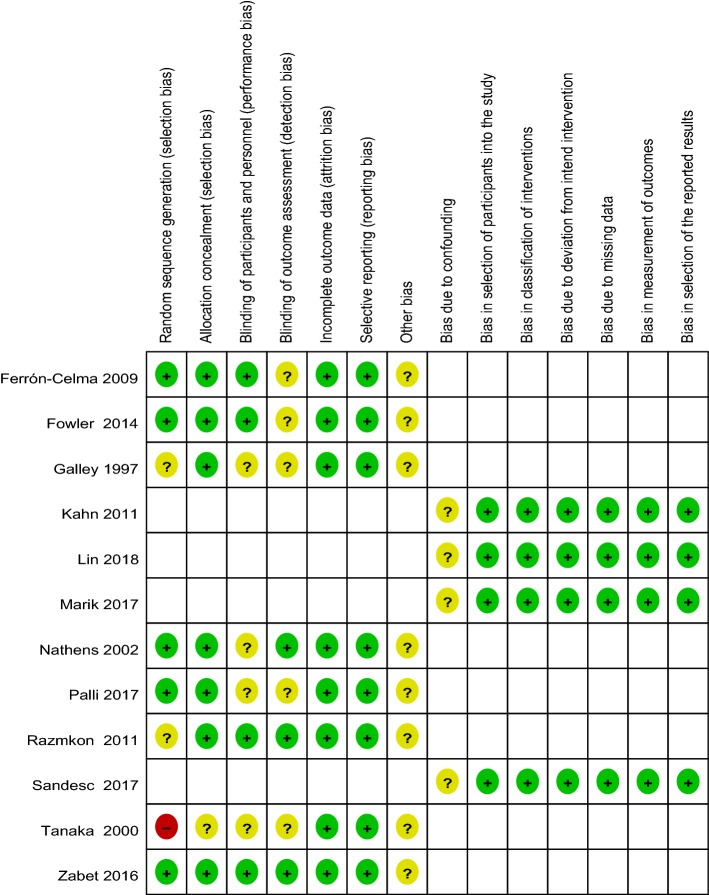
Risk of bias summary

The risk of bias is summarized in Fig. 2. No trials were considered low risk, and no specific details were used for assessment of the blinding outcomes. The trial performed by Tanaka and colleagues [18] despite being classed as a randomized study was deemed a high risk of bias as participants were allocated to groups according to months. Studies by Ramzkon et al. [21] and Galley et al. [24] lacked specific allocations and were classed as an unclear risk. In total, 11 trials were defined as an unclear risk and a single trial was deemed high risk.

### Meta-analysis results

#### Intravenous AA administration and ICU/hospital mortality

**Fig. 3:**
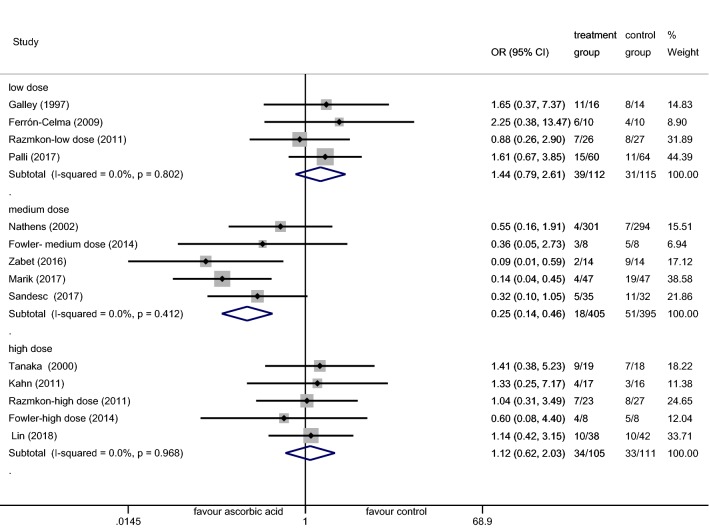
Forest plot of the effect of IV AA on mortality at the final follow-up when compared by the AA dose

**Fig. 4:**
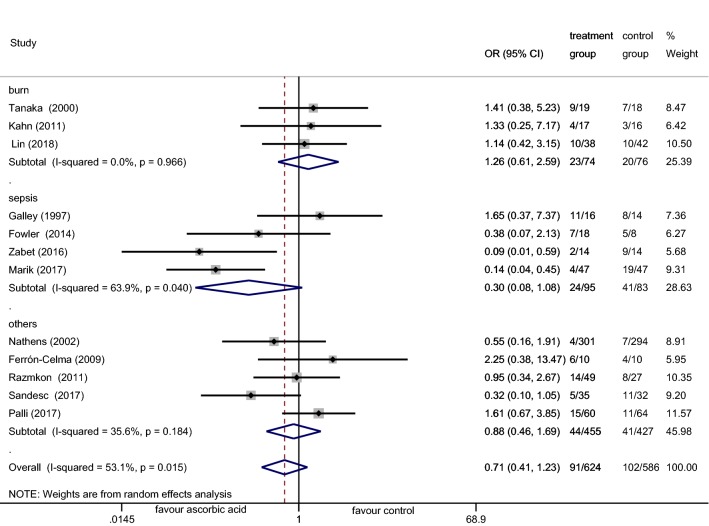
Forest plot of the effect of IV AA on mortality at the final follow-up when compared by the characteristics of patients

The MA included 12 trials comprising 1210 participants, of whom 624 received AA and 586 received placebo treatment. The analysis indicated that IV AA did not reduce the mortality of critically ill patients (OR 0.71; 95% CI (0.41–1.23); *p* = 0.219; *I*^2^ = 53.1%, Additional file 1: Fig. S1). A random (M-H heterogeneity) model was applied due to an *I*^2^ = 53.1%. Subgroup analysis suggested that the administration of AA alone or in combination with other antioxidant agents did not lower the mortality rates (OR 0.64; 95% CI (0.30–1.37); *p* = 0.253; *I*^2^ = 58.9% vs. OR 0.84; 95% CI (0.37–1.91); *p* = 0.678; *I*^2^ = 48.5%, Additional file 1: Fig. S1). As the dose of AA varied between the trials, subgroup analysis was performed. Doses < 3 g/d were defined as low, ≥ 10 g/d as high, and 3–10 g/d as medium. The medium AA dose was found to reduce the mortality of critically ill patients (OR 0.25; 95% CI (0.14–0.46); *p* < 0.001; *I*^2^ = 0.0%, Fig. 3), while neither low-dose AA (< 3 g/d) nor high-dose AA (≥ 10 g/d) influenced the mortality (OR 1.44; 95% CI (0.79–2.61); *p* = 0.234; *I*^2^ = 0.0% vs. OR 1.12; 95% CI (0.62–2.03); *p* = 0.700; *I*^2^ = 0.0%, Fig. 3). Due to homogeneous, a fixed Mantel–Haenszel model was applied. Subgroup analysis was adopted according to patient characteristics. The results revealed that in all patient conditions (including sepsis, burns, and others), AA did not decrease the mortality rates (OR 0.30; 95% CI (0.08–1.08); *p* = 0.066; *I*^2^ = 63.9% vs. OR 1.26; 95% CI (0.61–2.59); *p* = 0.538; *I*^2^ = 0.0% vs. OR 0.88; 95% CI (0.46–1.69); *p* = 0.706; *I*^2^ = 35.6%, Fig. 4). Sensitivity analysis was performed through the removal of each single trial and the reanalysis of the remaining trials according to sepsis subgroups. Upon excluding the Galley et al. study [24], the analysis was homogeneous and AA decreased the mortality of patients with sepsis (OR 0.16; 95% CI (0.07–0.37); *p* < 0.001; *I*^2^ = 0.0%, Additional file 2: Fig. S2).

#### Length of ICU and hospital stay

**Fig. 5:**
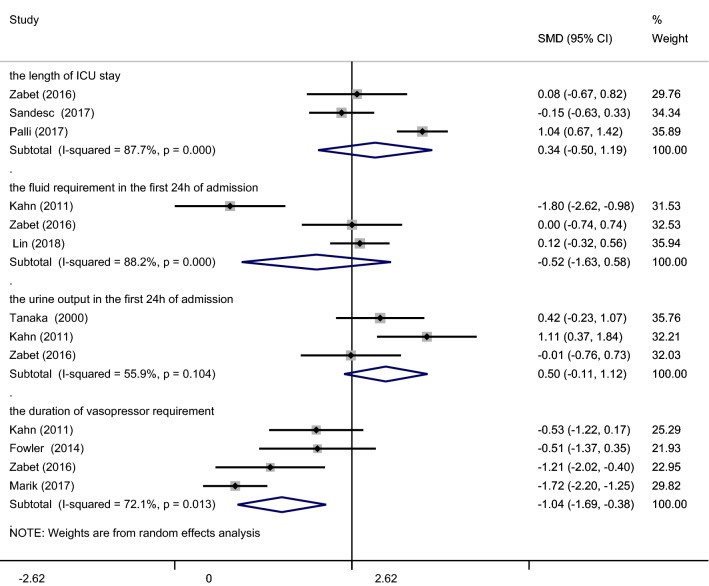
Forest plot of the effect of IV AA on the length of ICU stay, the fluid requirement or urine output in the first 24 h of admission, and the duration of vasopressor requirement

Three trials [16, 22, 23] compared the length of ICU stays between AA and control groups. AA was found to have no influence on the length of ICU residence (SMD = 0.34; 95% CI (− 0.50–1.19); *p* = 0.424; *I*^2^ = 87.7%, Fig. 5). As the *I*^2^ value = 87.7%, a random (M-H heterogeneity) model was applied. Sensitivity analysis was performed through the removal of each individual trial and through reanalysis of the remaining trials. When excluding the trial performed by Palli and colleagues [23], the analysis became homogeneous and the application of AA did not reduce the length of the ICU stay (SMD = − 0.08; 95% CI (− 0.49–0.32); *p* = 0.680; *I*^2^ = 0.0%).

**Fig. 6:**
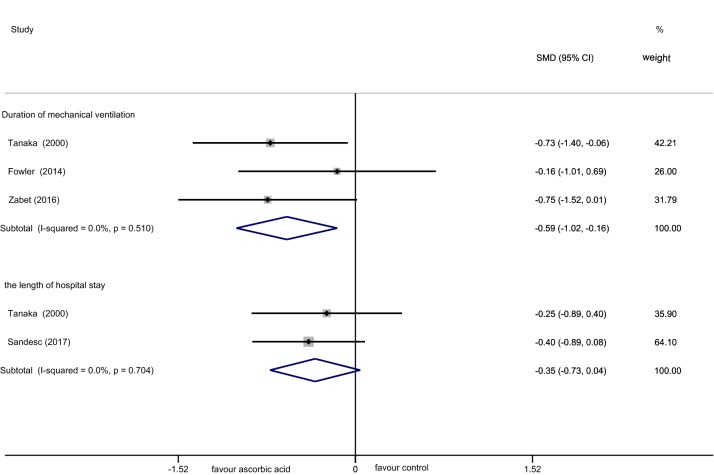
Forest plot of the effect of IV AA on the duration of mechanical ventilation and the length of hospital stay

Two trials [18, 22] were included to assess the impact of the length of hospital stay on which AA had no influence (SMD = − 0.35; 95% CI (− 0.73–0.04); *p* = 0.080; *I*^2^ = 0.0%, Fig. 6). As the data from these trials were homogeneous, a fixed Mantel–Haenszel model was employed.

### Fluid requirement, urine output and patients suffering from AKI

Our analysis among trials [16, 17, 19] suggested that AA intervention did not reduce the fluid requirement during the first 24 h of admission (SMD = − 0.52; 95% CI (− 1.63–0.58); *p* = 0.351; *I*^2^ = 88.2%, Fig. 5). A random (M-H heterogeneity) model was applied due to the *I*^2^ value = 88.2%. Sensitivity analysis was performed through the removal of each individual trial and reanalysis of the remaining trials. The results remained unaffected except for the removal of the Kahn et al. study [17] from which the analysis became homogeneous (SMD = 0.09; 95% CI (− 0.29–0.47); *p* = 0.650; *I*^2^ = 0.0%).

Upon AA administration, no increase in urine output was observed during the first 24 h of admission (SMD = 0.50; 95% CI (− 0.11–1.12); *p* = 0.110; *I*^2^ = 55.9%, Fig. 5). For heterogeneity analysis, a random (M-H heterogeneity) model was applied. For sensitivity analysis, removal of the Zabet et al. [16] study led to an increased urine output in response to AA (SMD = 0.72; 95% CI (0.23–1.21); *p* = 0.004; *I*^2^ = 47.1, Additional file 3: Fig. S3).

No differences were observed in the number of patients suffering from AKI (OR 1.40; 95% CI (0.74–2.63); *p* = 0.298; *I*^2^ = 38.5%, Additional file 4: Fig. S4). We applied a fixed Mantel–Haenszel model for this analysis [19, 20, 36].

### Duration of vasopressor requirement

The analysis consisted of 4 trials [15–17, 20] over which the duration of vasopressor requirement declined (SMD = − 1.04; 95% CI (− 1.69 to − 0.38); *p* = 0.002; *I*^2^ = 72.1%, Fig. 5). Upon consideration of the *I*^2^ value, we employed a random (M-H heterogeneity) model for the analysis. Upon sensitivity analysis, no significant changes were observed.

### Duration of mechanical ventilation

From the analysis among trials [14, 16, 18], a lower duration of mechanical ventilation (SMD = − 0.59; 95% CI (− 1.02 to − 0.16); *p* = 0.008; *I*^2^ = 0.0%, Fig. 6) was proposed. A fixed Mantel–Haenszel model was employed for this analysis.

### Publication bias

A Begg test was performed to assess the publication bias of the 11 included studies (*p* = 0.640). The analysis suggested that minimal publication bias occurred.

## Discussion

### Main Findings

#### IV AA administration and ICU/hospital mortality

Compared to other MAs [49, 50, 52], the major finding of this study was that IV medium doses (3–10 g/d) of AA were associated with decreased mortality, with neither low doses (< 3 g/d) nor high doses (≥ 10 g/d) having a significant impact. The MA conducted by Langlois and coworkers [52] included enteral and IV supplementation and suggested no association of AA with reduced mortality; subgroup analysis revealed that oral-enteral or parenteral, low or high administration of doses of AA (1.5 g/d as a boundary) did not significantly influence mortality.

Both dosing and bio-distribution data in humans suggest that pharmacological concentrations of AA are only attainable through IV administration due to the saturation of intestinal transporters (sodium-vitamin C transporter-1) [51]. Previous studies have shown that critically ill patients often have low AA plasma levels [4–7]. Furthermore, during the post-injury period, 2 days of 3000 mg/day AA significantly increase plasma AA concentrations [4]. Other studies [48] have shown that 10 g/d AA is associated with supranormal plasma concentrations. Considerable adverse effects have not been reported at high AA doses (66 mg/kg/h) [17–19]. In this study, we defined a dose lower than 3 g/d (not inclusive of 3 g/d) as low, higher than 10 g/d (inclusive of 10 g/d) as high, and 3–10 g/d as medium. Our analysis revealed that low-dose AA had little effect on mortality partly due to patients receiving IV AA ≤ 2 g/d (Table 1). Studies have shown that IV AA 2 g/d leads to only normal plasma concentrations [48]. In addition, AA is depleted by free iron, free radical scavengers in the plasma, and the destruction of oxidized AA and dehydroascorbic acid [43]. Thus, low AA doses do not influence mortality. For medium doses, improved patient prognosis was observed. Low levels of plasma AA in septic patients inversely correlated with the incidence of multiple organ failure [7], while medium doses restored the AA concentrations to normal plasma levels [48]. Additionally, studies performed by Straaten and coworkers [68] suggested that high-dose AA (3–6 g/d) decreases the formation of superoxide and peroxynitrite, bidirectly scavenges superoxides, augments antibacterial defenses, and protects against oxidative stress in critically ill patients. AA can counteract lipid peroxidative damage through the scavenging of oxygen-derived free radicals and the restoration of vascular function [53]. Counteracting oxidative stress represents a likely mechanism by which moderate AA reduces mortality. For high doses of AA, no loss of mortality was observed. Among the three trials [15–19, 21] participants suffering from burn injuries were recruited and the majority of deaths did not occur during resuscitation, but from subsequent infections [54]. In addition, patients in group B of the study by Razmkon and coworkers [21] received 10 g on the first day of admission which was repeated on day 4, followed by 4 g/d for the remaining 3 days. The final follow-up of mortality in these trials differed, partly accounting for the outcomes.

Through subgroup analysis of the patient characteristics, AA had no effect on sepsis or burns. For sepsis, heterogeneity was observed, mainly due to the Galley et al. [24] study. The Galley trial was performed in 1997, and the study protocols drastically differed from those of the more recent studies. When the Galley et al. trial was excluded, AA had a positive effect on mortality. For burn patients, AA had little influence. Further studies are warranted to dissect this relationship given that burn deaths mainly occur after resuscitation [54].

### Fluid requirement, urine output, and patients suffering from AKI

The results demonstrate that AA has little effect on fluid requirements or urine output during the initial 24 h, or the number of patients suffering from AKI. Upon consideration of the trial reported by Zabet et al. [16], although a significant increase in urine output during the first 24 h did not occur, a tendency toward increasing urine output over time was noted, which may have influenced the overall significance of the analysis. AA is hyperosmolar and is a risk factor for osmotic diuresis [17]. Despite the trial of Tanaka et al. [18], AA did not cause osmotic diuresis and no differences in the patients’ urine and serum osmolality were observed. In the trial of Kahn and colleagues [17], several patients showed signs of hypovolemia in the absence of decreased urine output, which was noted as a possible sign of osmotic diuresis. Other trials lacked data on urine output and osmotic diuresis and future studies should consider these parameters.

Previous studies suggested that following a severe burn injury, potent oxygen-free radicals (OFRs) are produced from the ischemia and reperfusion of burnt skin [60]. Animal studies reveal that antioxidant therapy through the administration of high-dose AA, an ORF scavenger [61], reduces post-burn lipid peroxidation [9], decreases vascular permeability [57], decreases burn and no-burn tissue edema [58], and reduces the requirement for resuscitation fluid [10, 18, 59]. However, this was not observed in this study. Possible reasons for these discrepancies include the varied initiation of treatment between the studies. In the study by Kahn et al. [17], treatment was initiated at 52 ± 26 min post-admission, while in study by Lin et al. [19] treatment was initiated at 4.01 ± 15 h. Zabet and coworkers [16] did not provide a specific treatment time. All the included studies were single-center, and the fluid levels received by the patients were variable. In the study by Lin and colleagues [19], although a reduction in fluid requirement was noted, both groups had higher weights and had a higher  % of total body surface area (TBSA) of burn injuries than would be estimated based on the Parkland formula. The trials lacked data on fluid requirement for longer periods and future studies should consider these parameters.

It has been reported that AA promotes acute renal failure [62, 63]. In patients with renal impairment who received high AA doses, increased levels of oxalate (typically excreted by the kidney) were observed in conjunction with crystallization, leading to impaired kidney function [64, 65]. In this study, effects on patients suffering from AKI were not observed partly due to the AA dose. In studies by Marik and Nathens [20, 36], AA was administrated at doses of 6 g/d and 3 g/d, respectively, which was considered medium doses, while Lin and colleagues [19] assessed the effects of 66 mg/kg/h AA. These trials included lacked data on time span during which AKI was measured. This may account for the overall outcome observed.

### Duration of vasopressor requirement

From our studies, AA significantly reduced the duration of vasopressor requirement. AA is an essential cofactor for the copper-containing enzyme dopamine β-hydroxylase during catecholamine (dopamine, norepinephrine, and epinephrine) synthesis [55]. It was found that IV AA improved cardiovascular function and decreased the requirement for catecholamine in a patient with septic shock [56]. Daniel and colleagues [69] stated that AA was a cofactor during collagen synthesis that was required to support cardiovascular functions. Additionally, Carr et al. [13] found that AA increases vasopressor sensitivity.

### Duration of mechanical ventilation

A decreased duration of mechanical ventilation upon AA administration was observed, but whether this reflected improved pulmonary function was uncertain. Tanaka et al. [18] commented that fewer days of mechanical ventilation and improved early respiratory function were associated with fluid reduction. The opposite scenario of fluid requirement was observed in this study. Animal models of sepsis suggest that IV AA attenuates proinflammatory and procoagulant states, reducing lung vascular injury [11] and oxidative stress, induced histopathological alterations, thus improving pulmonary function [66]. Only the Tanaka et al. [18] study provided pulmonary function measurements. Future studies should consider these parameters.

### Comparison with other studies

The major strength of this MA was the investigation of how different AA doses contribute to the clinical outcomes of patients with critical illness. We compared the effects of AA alone or in combination with other agents for its effects on mortality and compared the effects of AA on different patient characteristics (sepsis, burn, or others). Such studies have not been previously performed. Furthermore, a varied mix of medical, surgical, and burn injury patients was included, and each trial equally contributed to the final study outcomes.

### Limitations

This MA had several weaknesses that should be noted. Firstly, 12 trials were included of which 8 were RCTs and 4 were retrospective trials. The study sizes were relatively small, and all trials were single-center. The included participants varied in terms of medical, surgical, and burn status characteristics, which may have led to study heterogeneity. Secondly, the initiation of treatment, the duration of therapy, and follow-up varied between the trials, which may have influenced the outcomes. The adequate dosing of antioxidants, administration routes, timings, the initiation of treatment, the duration of therapy, and the role of single versus combination therapy still requires clarification in future studies [67].

## Conclusion

Based on the current available evidence, the IV administration of AA can narrow the duration of vasopressor requirement and mechanical ventilation, but plays little role in fluid requirement or urine output during the first 24 h of admission, or the number of patients suffering from AKI, as well as the length of ICU or hospital stay. Furthermore, medium dose (3–10 g/d) AA has a positive role in mortality, which is not achieved by low (< 3 g/d) or high doses (≥ 10 g/d). However, given the limitations of the study combined with the heterogeneity, further studies are required to clarify the role of AA during the management of critically ill patients.

## Additional files

**Additional file 1.** Forest plot of the effect of IV AA on mortality at the final follow-up when compared by administration of AA alone or in combination with other antioxidant agents.

**Additional file 2.** Forest plot of the effect of IV AA on mortality at the final follow-up in the subgroup of sepsis by removing the trial of Galley.

**Additional file 3.** Forest plot of the effect of IV AA on the urine output in the first 24 h of admission when removing the trials of Zabet [16].

**Additional file 4.** Forest plot of the effect of intravenous ascorbic acid administration on the number of patients suffered from AKI.

## Data Availability

Not applicable.

## Abbreviations

AA: ascorbic acid
MA: meta-analysis
IV: intravenous
AKI: acute kidney injury
ICU: intensive care unit
ROS: reactive oxygen species
RCTs: randomized controlled trials
Ors: odds ratios
CIs: confidence intervals
OFRs: oxygen-free radical
TBSA: total body surface area of burn injury
