# Supplementary material for: Effects of different ascorbic acid doses on the mortality of critically ill patients: a meta-analysis
Source: Ann Intensive Care. 2019 May 20;9:58. doi: 10.1186/s13613-019-0532-9 (PMC6527630; doi:10.1186/s13613-019-0532-9)
Supplement: Supplementary file 1 — Additional file 1. Forest plot of the effect of IV AA on mortality at the final follow-up when compared by administration of AA alone or in combination with other antioxidant agents. [file 13613_2019_532_MOESM1_ESM.pdf]

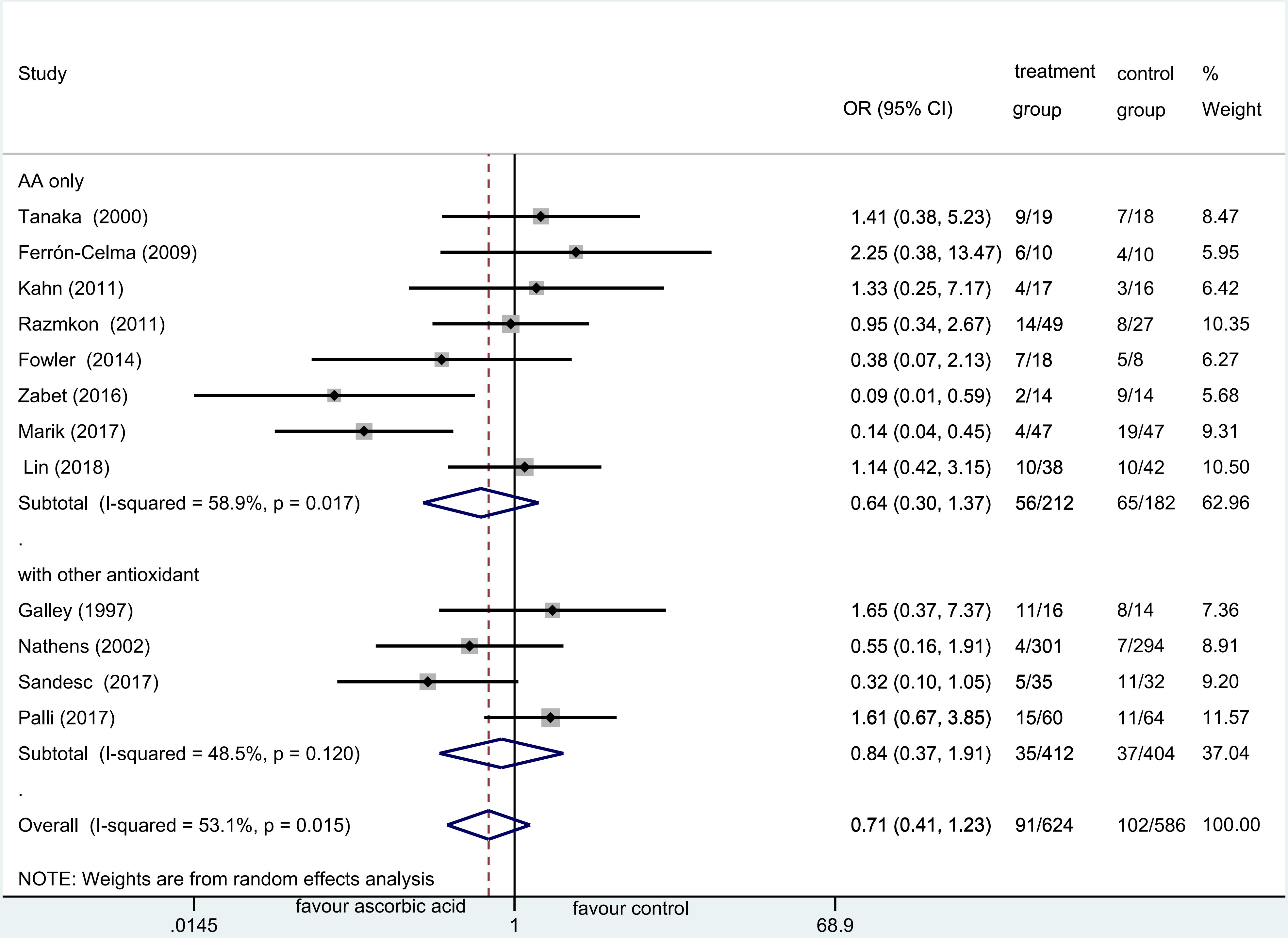

Fig S1: Forest plot of the effect of IV AA on mortality at the final follow-up when compared by administration of AA alone or in combination with other antioxidant agents
