# Supplementary material for: Effects of different ascorbic acid doses on the mortality of critically ill patients: a meta-analysis
Source: Ann Intensive Care. 2019 May 20;9:58. doi: 10.1186/s13613-019-0532-9 (PMC6527630; doi:10.1186/s13613-019-0532-9)
Supplement: Supplementary file 2 — Additional file 2. Forest plot of the effect of IV AA on mortality at the final follow-up in the subgroup of sepsis by removing the trial of Galley. [file 13613_2019_532_MOESM2_ESM.pdf]

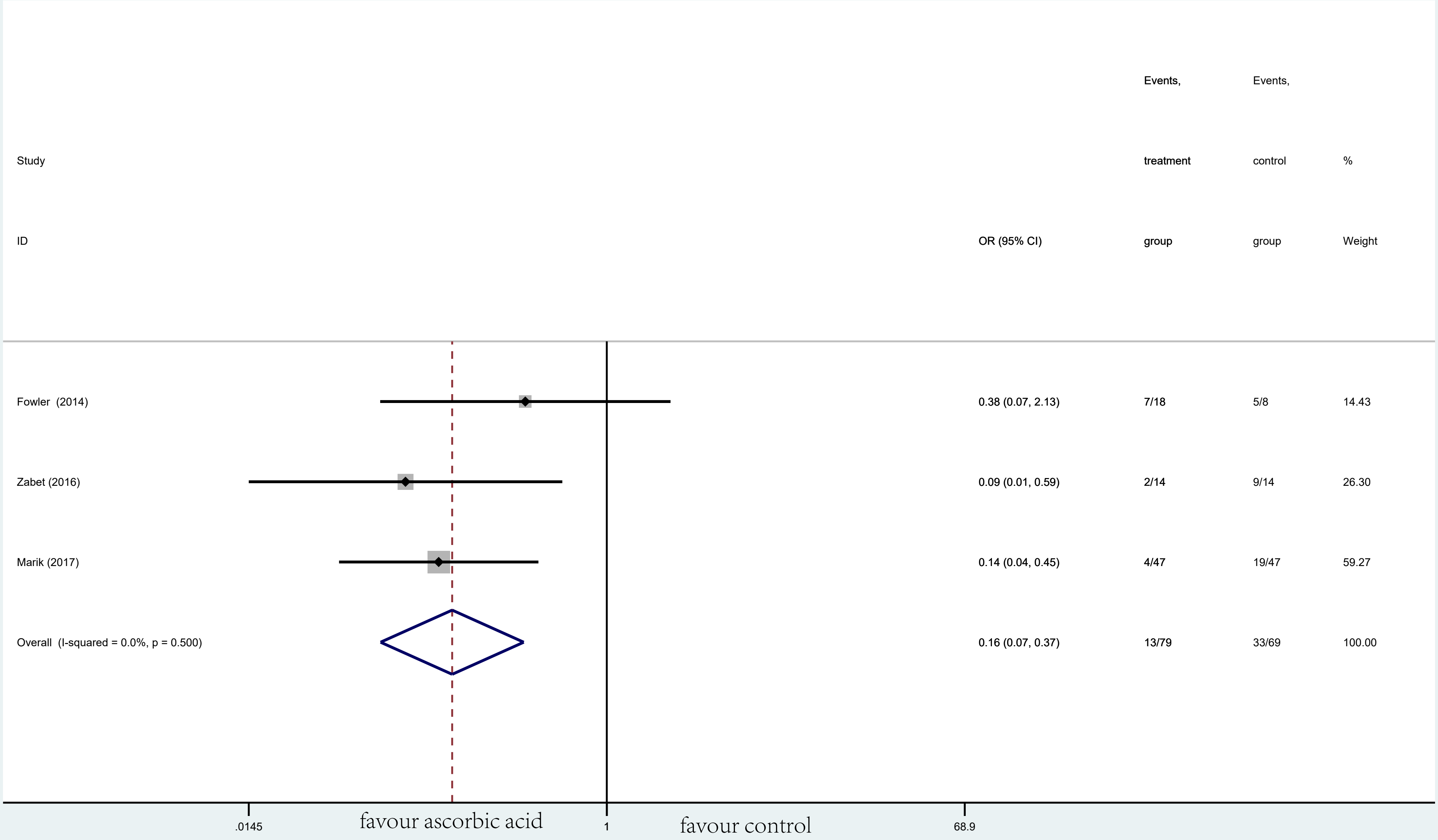

Fig S2: Forest plot of the effect of IV AA on mortality at the final follow-up in the subgroup of sepsis by removing the trial of Galley.
