# Supplementary material for: Effects of different ascorbic acid doses on the mortality of critically ill patients: a meta-analysis
Source: Ann Intensive Care. 2019 May 20;9:58. doi: 10.1186/s13613-019-0532-9 (PMC6527630; doi:10.1186/s13613-019-0532-9)
Supplement: Supplementary file 3 — Additional file 3. Forest plot of the effect of IV AA on the urine output in the first 24 h of admission when removing the trials of Zabet [16]. [file 13613_2019_532_MOESM3_ESM.pdf]

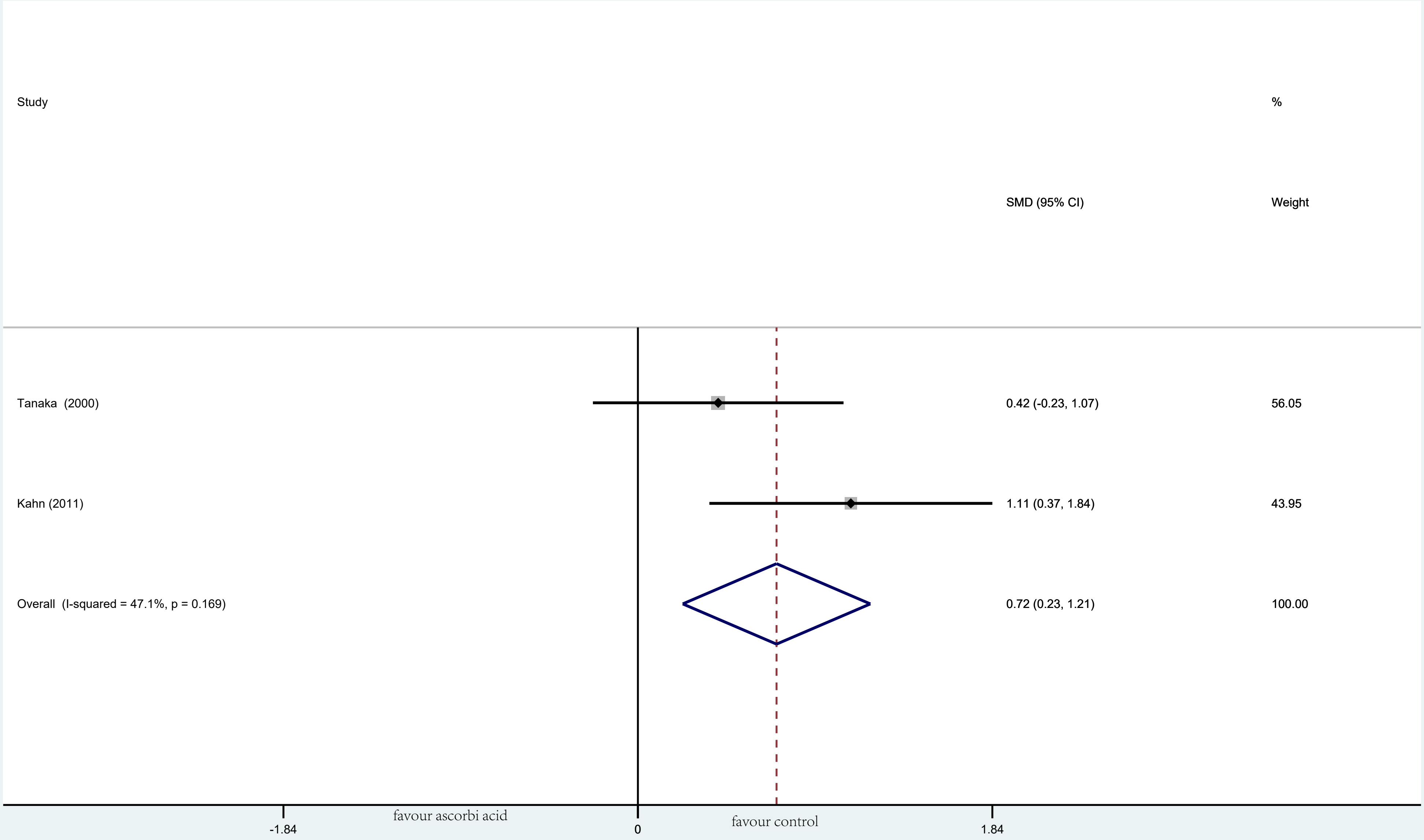

Fig S3: Forest plot of the effect of IV AA on the urine output in the first 24 hours of admission when removing the trials of Zabet[16]
