# Supplementary material for: Effects of different ascorbic acid doses on the mortality of critically ill patients: a meta-analysis
Source: Ann Intensive Care. 2019 May 20;9:58. doi: 10.1186/s13613-019-0532-9 (PMC6527630; doi:10.1186/s13613-019-0532-9)
Supplement: Supplementary file 4 — Additional file 4. Forest plot of the effect of intravenous ascorbic acid administration on the number of patients suffered from AKI. [file 13613_2019_532_MOESM4_ESM.pdf]

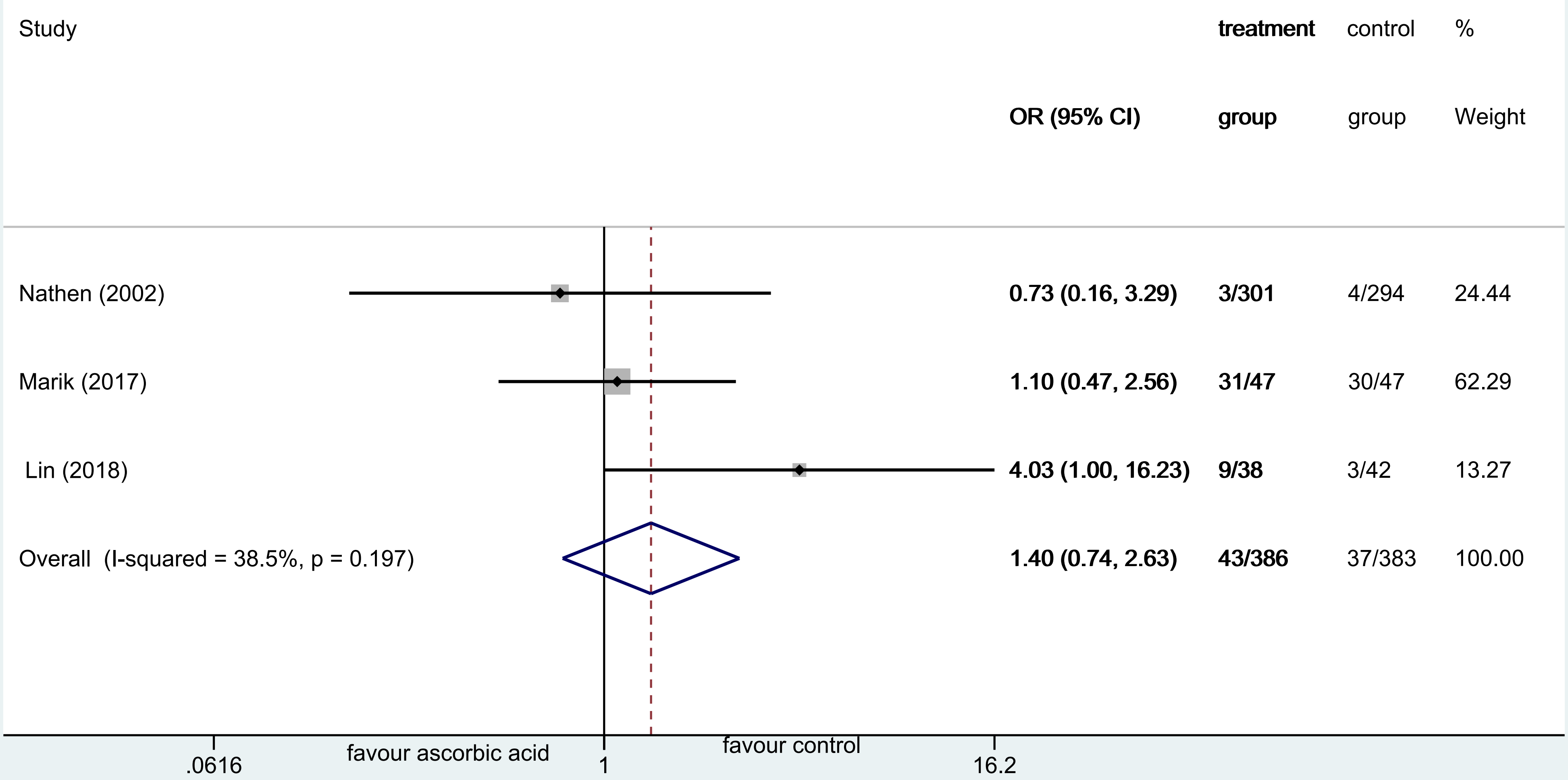

Fig S4: Forest plot of the effect of intravenous ascorbic acid administration on the number of patients suffered from AKI.
